# *ADRB3* Gene Trp64Arg Polymorphism and Essential Hypertension: A Meta-Analysis Including 9,555 Subjects

**DOI:** 10.3389/fgene.2018.00106

**Published:** 2018-04-04

**Authors:** Yan-yan Li, Xin-zheng Lu, Hui Wang, Yan-hong Zhou, Xin-xing Yang, Hong-yu Geng, Ge Gong, Hyun Jun Kim

**Affiliations:** ^1^Department of Geriatrics, First Affiliated Hospital of Nanjing Medical University, Nanjing, China; ^2^Clinical Research Center, First Affiliated Hospital of Nanjing Medical University, Nanjing, China; ^3^Department of Cardiology, First Affiliated Hospital of Nanjing Medical University, Nanjing, China; ^4^Department of Intensive Care Unit, Baoding First Central Hospital, Baoding, China; ^5^Department of Geriatrics, Nanjing General Hospital, Nanjing, China; ^6^Department of Physiology, University of Cincinnati, Cincinnati, OH, United States

**Keywords:** *ADRB3*, Trp64Arg, polymorphism, essential hypertension, rs4994

## Abstract

**Background:** Presence of the β*3-Adrenergic receptor (ADRB3)* gene Trp64Arg (T64A) polymorphism may be associated with an increased susceptibility for essential hypertension (EH). A clear consensus, however, has yet to be reached.

**Objective and methods:** To further elucidate the relationship between the *ADRB3* gene Trp64Arg polymorphism and EH, a meta-analysis of 9,555 subjects aggregated from 16 individual studies was performed. The combined odds ratios (ORs) and their corresponding 95% confidence intervals (CI) were evaluated using either a random or fixed effect model.

**Results:** We found a marginally significant association between *ADRB3* gene Trp64Arg polymorphism and EH in the whole population under the additive genetic model (OR: 1.200, 95% CI: 1.00–1.43, *P* = 0.049). Association within the Chinese subgroup, however, was significant under allelic (OR: 1.150, 95% CI: 1.002–1.320, *P* = 0.046), dominant (OR: 1.213, 95% CI: 1.005–1.464, *P* = 0.044), heterozygous (OR: 1.430, 95% CI:1.040–1.970, *P* = 0.03), and additive genetic models (OR: 1.280, 95% CI: 1.030–1.580, *P* = 0.02). A significant association was also found in the Caucasian subgroup under allelic (OR: 1.850, 95% CI: 1. 260–2.720, *P* = 0.002), dominant (OR: 2.004, 95% CI: 1.316–3.052, *P* = 0.001), heterozygous (OR: 2.220, 95% CI: 1.450–3.400, *P* = 0.0002), and additive genetic models (OR: 2.000, 95% CI: 1. 330–3.010, *P* = 0.0009).

**Conclusions:** The presence of the *ADRB3* gene Trp64Arg polymorphism is positively associated with EH, especially in the Chinese and Caucasian population. The Arg allele carriers of *ADRB3* gene Trp64Arg polymorphism may be at an increased risk for developing EH.

## Introduction

Essential hypertension (EH) is a frequently-occurring condition that is associated with an increased risk of atherosclerosis, stroke, heart failure, and kidney failure (Ding et al., 2018; Omisore et al., 2018; Owolabi et al., 2018). These two factors combined, EH presents a significant barrier to health. The genetic epidemiological studies show that EH is a hereditary disease caused by the interplay of multiple genes and the environment. To explore the pathogenesis of EH, researchers are employing techniques from molecular biology to analyze potential genes that may act as risk factors and many believe the *ADRB3* to be a strong candidate (Kawaguchi et al., 2006).

The β3-adrenergic receptor (β3-AR, ADRB3) is an important component of sympathetic nervous system that primarily mediates lipolysis and thermogenic function. When the receptor is excited, lipolysis is increased locally. Mutation of the *ADRB3* gene can either reduce gene expression on the cytomembrane gene expression or cause an abnormal protein conformation that impairs ADRB3 function. The reduced cellular signal transduction from the repair which prevents adipose tissue heat production and decomposition can contribute to the pathogenesis of multiple conditions, including EH, diabetes mellitus (DM), and obesity.

Human *ADRB3* gene, located in 8p11.1–p12, contains 2 exons and 1 intron and encodes 408 amino acids. Mutation of the thymine in the 190th place to cytosine changes the 64th amino acid tryptophan (Trp) into an arginine (Arg) residue in the first intracellular loop of the ADRB3 receptor. This mutation is associated with a ten-fold decrease in adipocyte receptor sensitivity. The Trp64Arg (rs4994) variant is located in the 1st cellular inner ring which is crucial for both the movement and effector function of this receptor. The *ADRB3* Trp64Arg variant acts as a “thrifty” gene and has already been associated with obesity, DM, and insulin resistance (IR) (Hoffstedt et al., 1999).

Though many studies on the relationship between *ADRB3* gene Trp64Arg polymorphism and EH have been performed, a consensus has yet to be reached. In 2001, Wang et al. found a positive association in a Chinese Beijing population with the 64Arg allele acting as a risk factor for EH (Wang Y. et al., 2001). In 2000, Ringel et al. found the same result in a German population (Ringel et al., 2000). However, other papers reported no significant association in a Beijing and Hong Kong Chinese population (Thomas et al., 2000; Liu et al., 2015). While in 2005, Masuo et al. found the 64Trp allele is candidate allele for EH in a Japanese population (Masuo et al., 2005).

To explore the association of *ADRB3* gene Trp64Arg polymorphism and EH, we conducted a meta-analysis of 5,088 EH patients and 4,467 controls from 16 separate studies to evaluate the relationship of *ADRB3* gene Trp64Arg polymorphism and EH (Supplementary Table 1).

## Materials and methods

### Publication search and inclusion criteria

The electronic databases PubMed, the Wan Fang database, the VIP database, the China National Knowledge Infrastructure, the China Biological Medicine Database, Embase, and the Web of Science were searched using the terms “β*3-Adrenergic receptor*,” “*ADRB3*,” “Trp64Arg,” “essential hypertension” and “polymorphism.” The publication years ranged from 1997 to 2015 with the most recent update occurring on March 3, 2018.

The selected studies evaluated on the following criteria: (a) Evaluation of the relationship of EH and *ADRB3* gene Trp64Arg polymorphism. (b) EH diagnosis by a systolic BP no <140 mmHg and a diastolic BP no <90 mmHg measured three times on different days after the secondary hypertension was excluded. (c) The included studies should be case-controlled or officially published cohort studies. (d) The genotype number of *ADRB3* gene Trp64Arg polymorphism in the control group should follow the Hardy-Weinberg equilibrium (HWE).

### Data extraction

The data was extracted using a standardized protocol carried out by three investigators. Two were responsible for searching for duplicates among individual while the third one acting as an arbiter to settle differences. The studies violating the inclusion criteria, published repeatedly, or providing inadequate data were rejected. Similar data appeared in different papers by the same author group was adopted only once. The following items as publication year, ethnicity, region, the first author's name, genotyping method, matching criteria, genotype number, and total number of cases and controls should be included in the listed data table.

### Statistical analyses

Six genetic models were used in the current meta-analysis: allelic (A allele distribution frequency), recessive (AA vs. TA+TT), dominant (AA+TA vs. TT), homozygous (AA vs. TT), heterozygous (TA vs. TT), and additive (T vs. A) genetic models. The odds ratios (ORs) and their corresponding 95% confidence intervals (CIs) were used to compare the relationship of *ADRB3* gene Trp64Arg polymorphism and EH. The heterogeneity (HTG) among the individual studies was calculated by using a chi-square-based Q-tests with the significance set at *P* < 0.05 level (Cochran, 1968). The random-effects model (DerSimonian and Laird method) would be adopted when the HTG existed among the different studies (DerSimonian and Laird, 1986). If not, the fixed-effect model would be used (the Mantel–Haenszelmethod) (Mantel and Haenszel, 1959). The pooled OR was assessed by a *Z*-test with significance set at *P* < 0.05.

The HWE was evaluated by using Fisher's exact test with significance set at *P* < 0.05 level. Potential publication bias was assessed using a funnel plot. The funnel plot symmetry was evaluated by using Egger's linear regression test on the natural logarithm scale of the OR with significance set at *P* < 0.05 level (Egger et al., 1997). The statistical analyses were performed by using Stata 12.0 (StataCorp, College Station, TX, USA) and Revman 5.0 software.

## Results

### Studies and populations

Information was extracted from a total of 5,088 EH cases and 4,467 controls (Table 1) (Fujisawa et al., 1997; Baba et al., 1998; Tonolo et al., 1999; Ringel et al., 2000; Thomas et al., 2000; Wang Y. et al., 2001; Ding et al., 2002, 2009; Liang et al., 2004; Masuo et al., 2005; Tan et al., 2005; Guo et al., 2006; Chen et al., 2008; Zhang et al., 2012; Wang et al., 2013; Liu et al., 2015; Supplementary Table 2). Twenty-five papers were obtained through the search process, among which 16 papers were included for the present meta-analysis. Among the nine excluded studies, three papers were of review character and four papers deviated from the HWE (Wang M. Q. et al., 2001; Liu et al., 2006, 2013; Niu et al., 2007). Two papers had nothing with the *ADRB3* gene Trp64Arg polymorphism or EH.

**Table 1:** Characteristics of the investigated studies of the association between β*3-Adrenergic receptor (ADRB3)* gene Trp64Arg polymorphism and essential hypertension (EH).

**Author/Year**	**Region**	**Genotype**	**EH**			**Control**			**Matching criteria**	**SAMPLE size (EH/control)**
			**TT**	**TA**	**AA**	**TT**	**TA**	**AA**		
Chen et al., 2008	China (Fujian)	PCR-RFLP	266	96	10	233	67	6	Sex, Cr, ethnicity	372/306
Ding et al., 2002	China (Jiangsu)	PCR-RFLP	22	12	4	53	16	3	Ethnicity	38/72
Guo et al., 2006	China (Xinjiang)	PCR-RFLP	149	39	1	103	30	0	Ethnicity	189/133
Liang et al., 2004	China (Liaonig)	PCR-RFLP	94	40	10	119	42	7	Ethnicity	144/168
Liu et al., 2015	China (Beijing)	TaqMan	637	265	18	488	180	13	Age, sex, ethnicity	920/681
Tan et al., 2005	China (Guangdong)	PCR-RFLP	99	25	4	47	9	2	Ethnicity	128/58
Thomas et al., 2000	China (Hongkong)	PCR-RFLP	286	70	7	81	28	3	Sex, ethnicity	363/212
Wang et al., 2013	China (Beijing)	TaqMan	584	258	11	474	169	19	Age, sex, ethnicity	865/665
Wang Y. et al., 2001	China (Beijing)	PCR-RFLP	39	36	4	34	7	1	Age, sex, BP, ethnicity	179/42
Zhang et al., 2012	China (Neimenggu)	PCR-RFLP	71	26	5	72	19	2	Age, BMI, BP, ethnicity	102/91
Baba et al., 1998	Japan	PCR-RFLP	28	9	0	30	14	2	Age, sex, BMI, ethnicity	37/46
Ding et al., 2009	Japan	PCR-RFLP	860	408	60	904	433	49	Age, sex, BP, ethnicity	1328/1386
Fujisawa et al., 1997	Japan	PCR-RFLP	68	31	2	48	23	2	Cholesterol, ethnicity	101/73
Masuo et al., 2005	Japan	TaqMan	35	5	1	71	44	2	Age, smokers, ethnicity	41/117
Ringel et al., 2000	German	PCR-RFLP	150	30	0	214	23	0	Age, BMI, ethnicity	180/237
Tonolo et al., 1999	Italy	PCR-RFLP	184	29	0	262	19	0	Age, Cholesterol, ethnicity	213/281

*HDL, high density lipoprotein-cholesterol; BP, blood pressure; TG, triglyceride; LDL, low density lipoprotein-cholesterol; BMI, body mass index; PCR-LDR, Polymerase chain reaction-restriction fragment length polymorphism; Case-control study design was adopted in the above studies*.

### Pooled analyses

**Figure 1:**
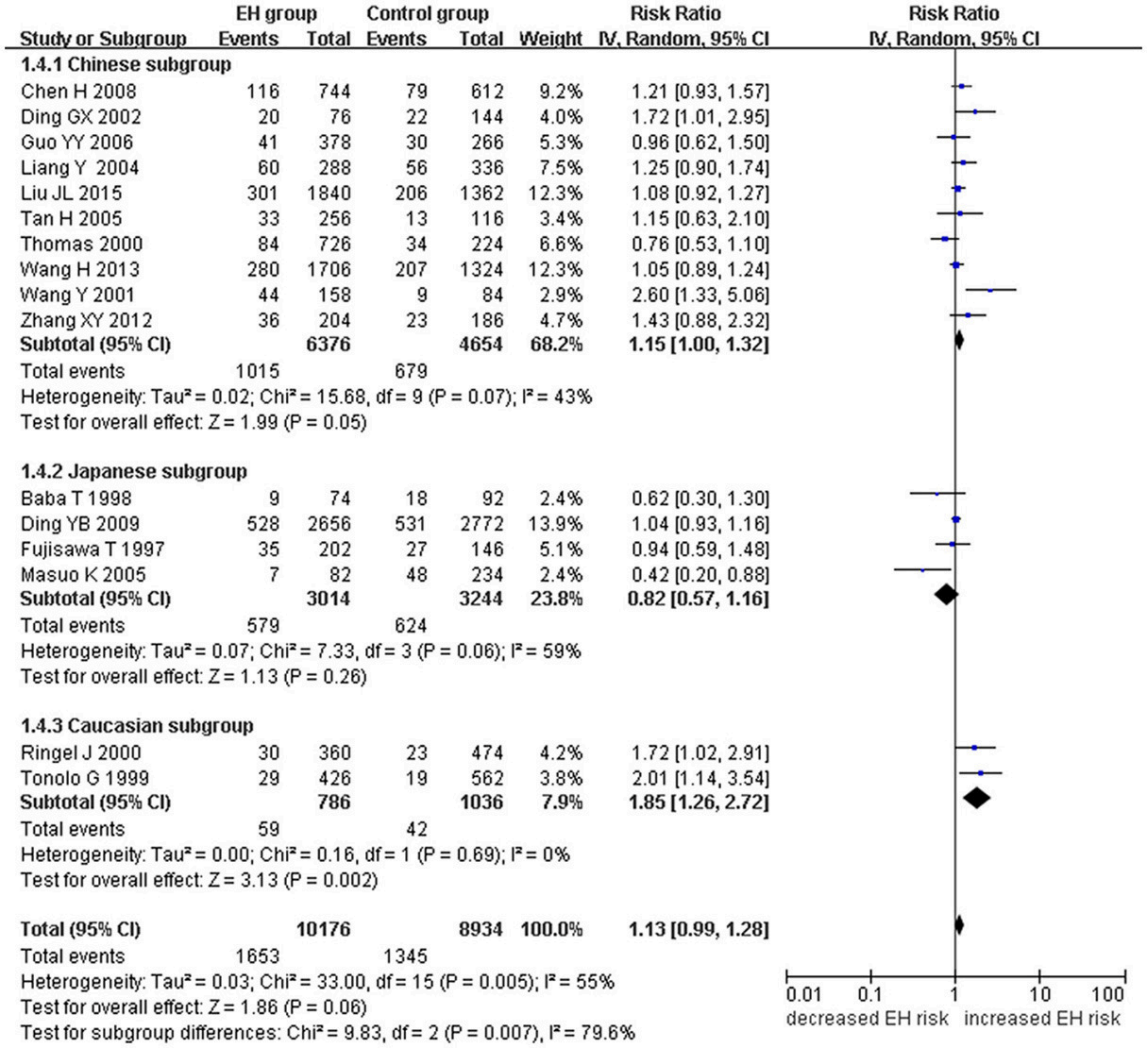
Forest plot of EH associated with *ADRB3* gene Trp64Arg polymorphism under an allelic genetic model (distribution of Arg allelic frequency of *ADRB3* gene Trp64Arg polymorphism).

**Figure 2:**
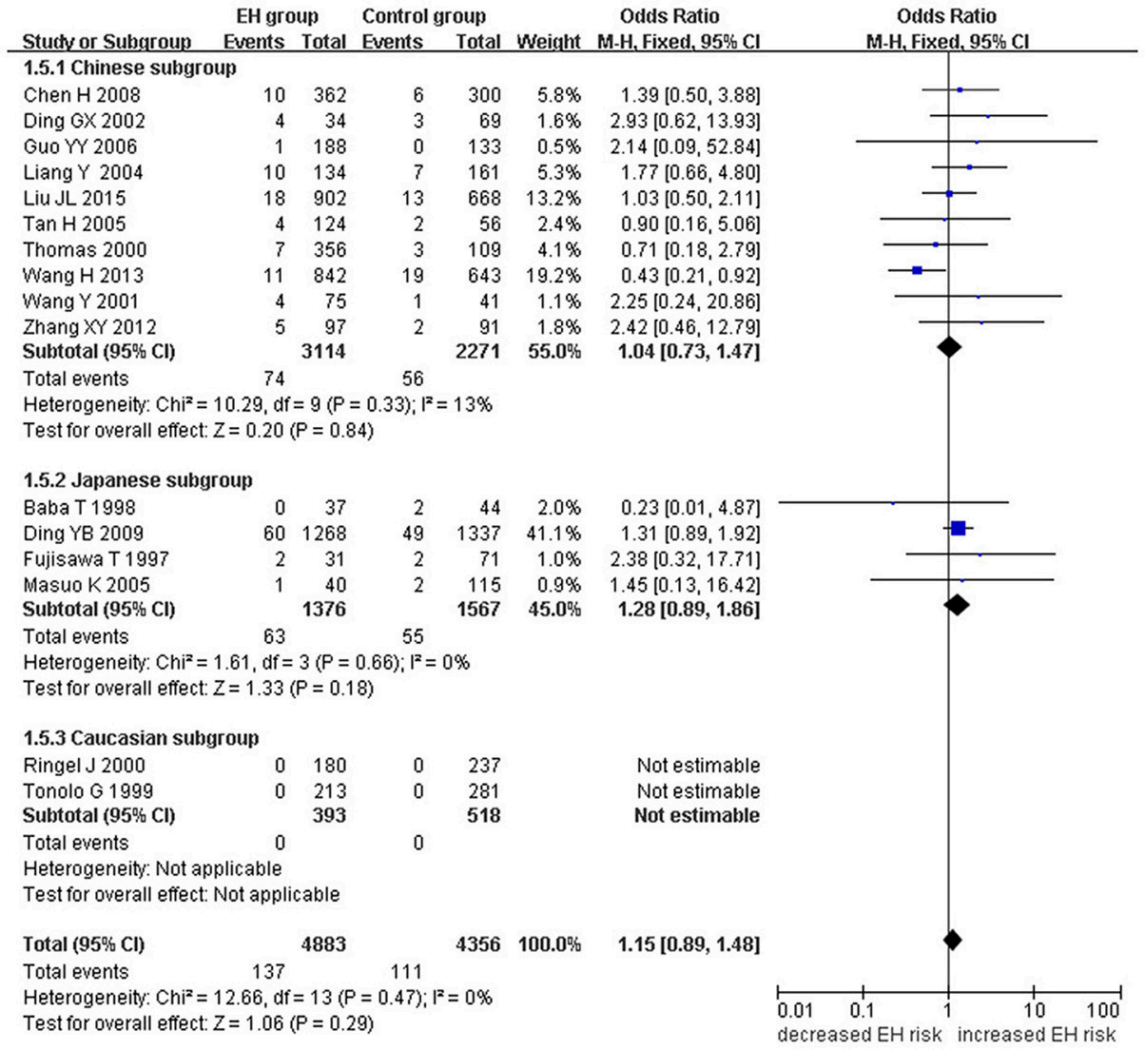
Forest plot of EH associated with *ADRB3* gene Trp64Arg polymorphism under a recessive genetic model (AA vs. TA+TT).

**Figure 3:**
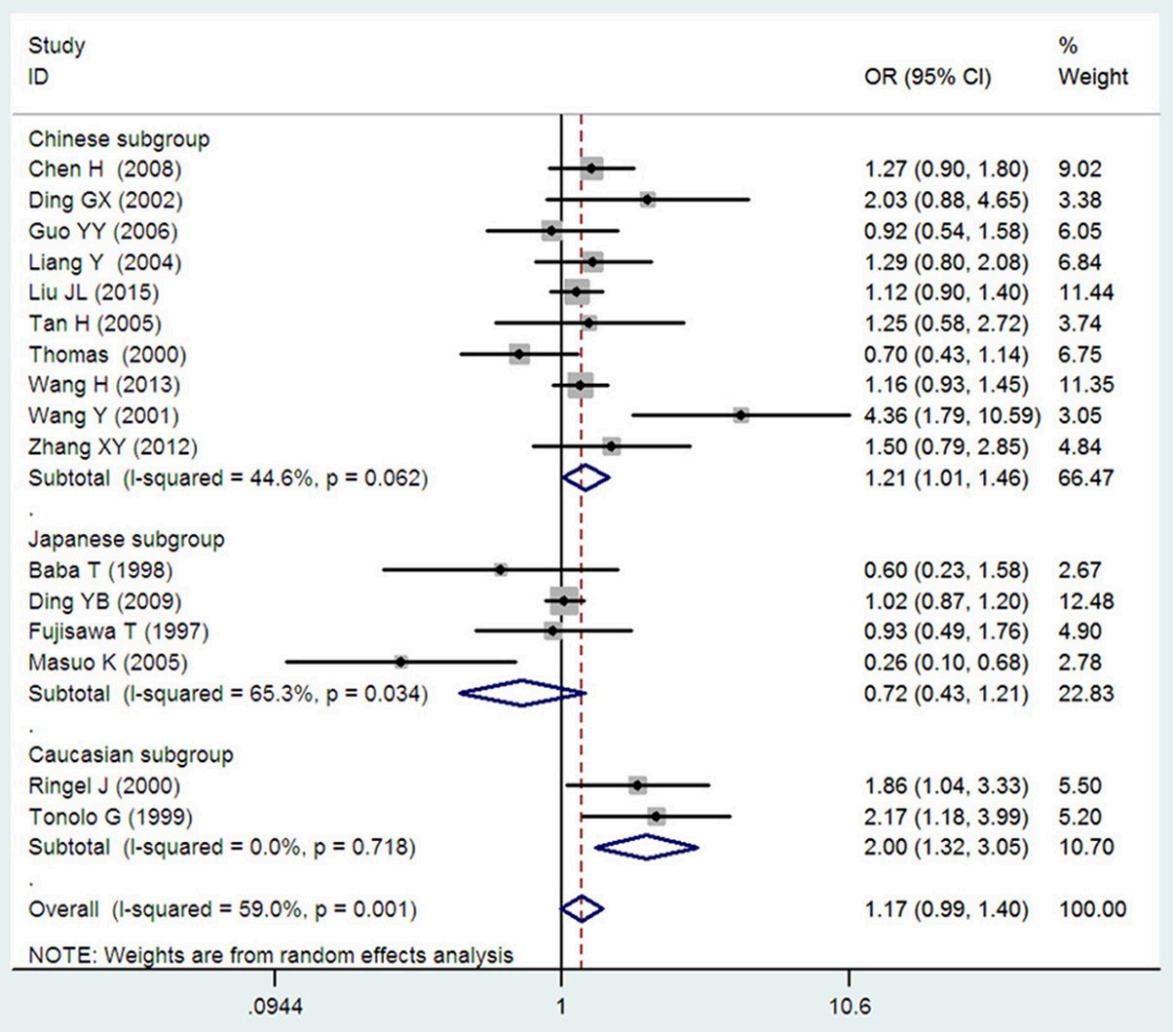
Forest plot of EH associated with *ADRB3* gene Trp64Arg polymorphism under a dominant genetic model (AA+TA vs. TT).

**Figure 4:**
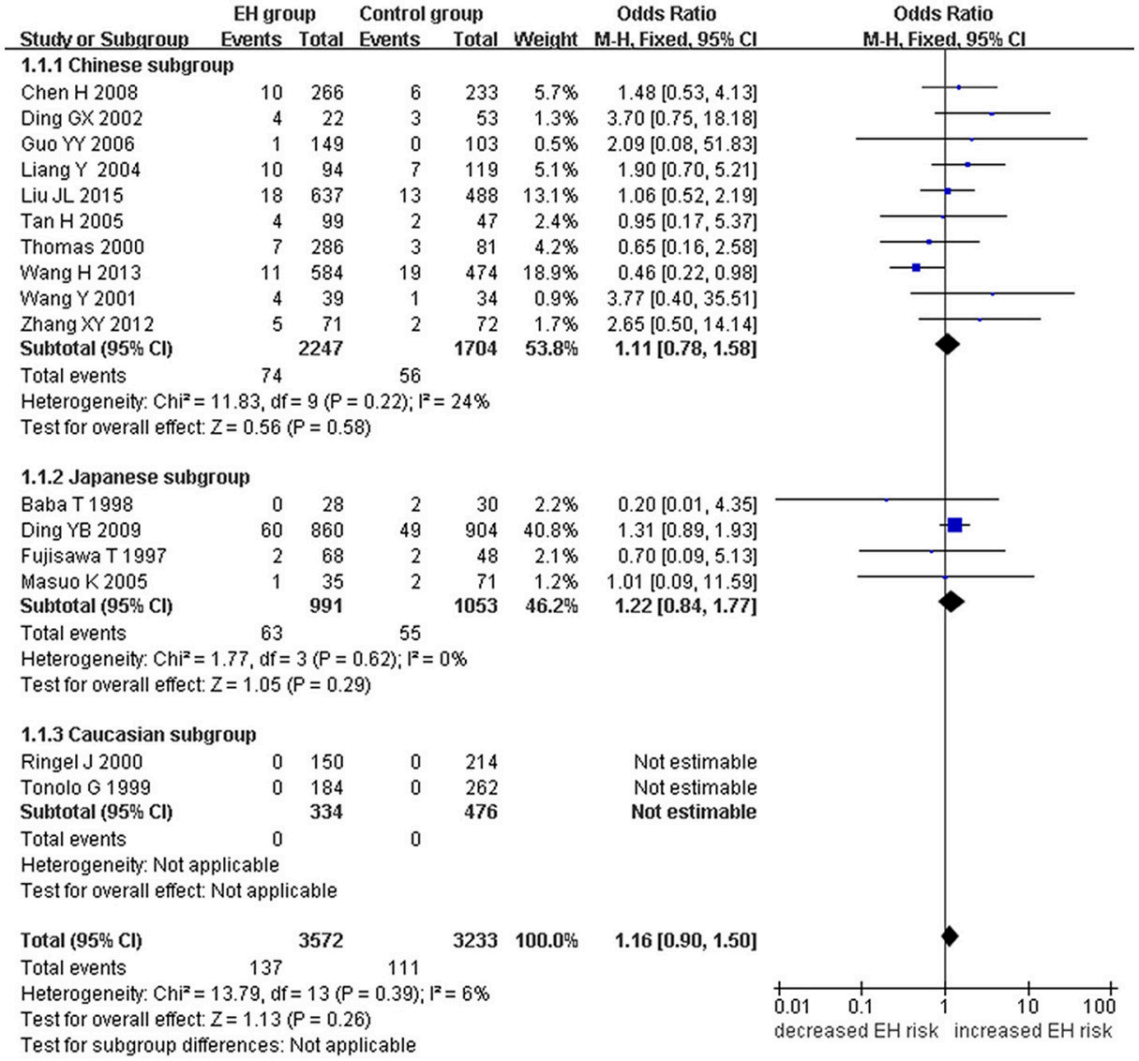
Forest plot of EH associated with *ADRB3* gene Trp64Arg polymorphism under a homozygous genetic model (AA vs. TT).

**Figure 5:**
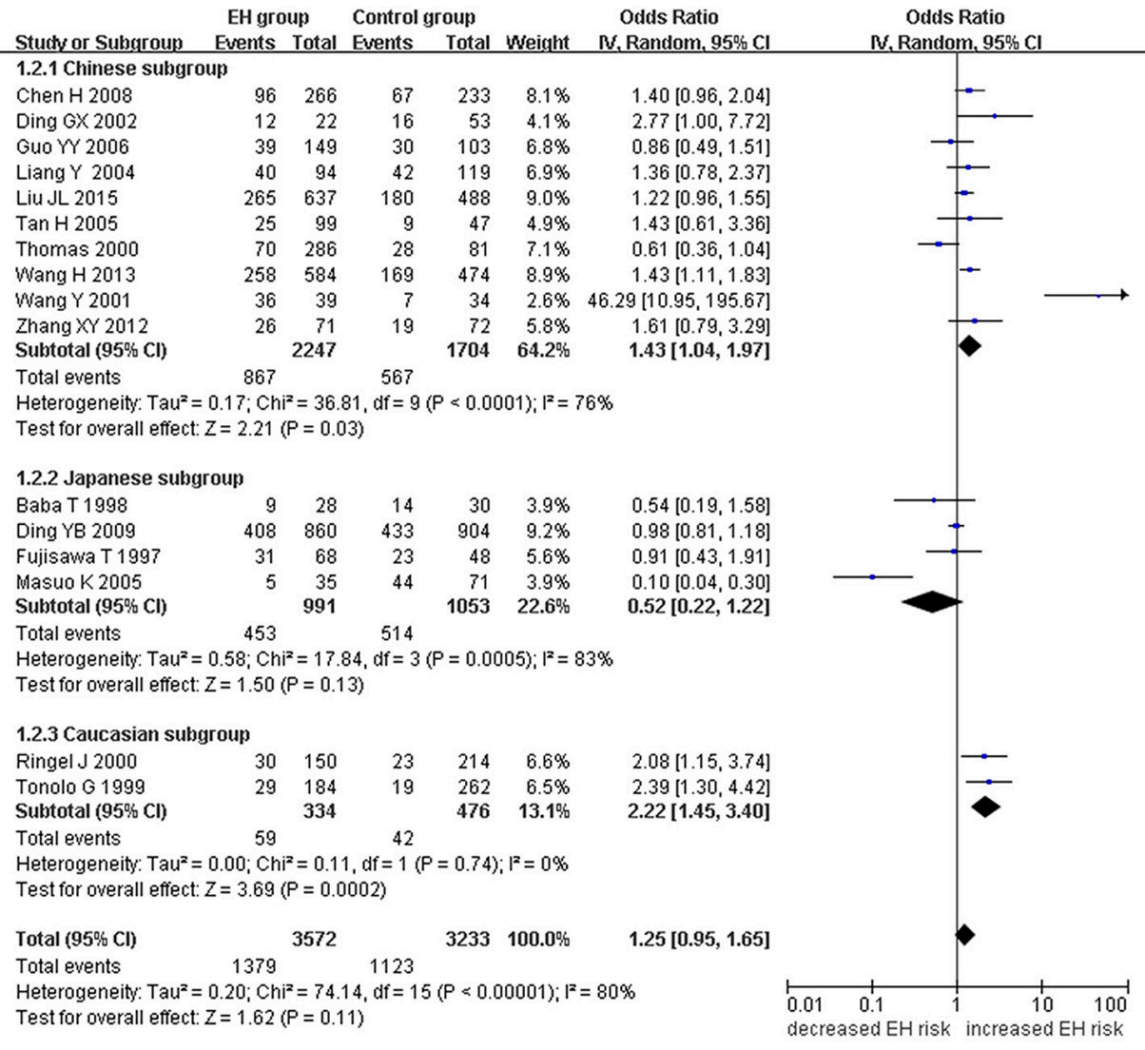
Forest plot of EH associated with *ADRB3* gene Trp64Arg polymorphism under a heterozygous genetic model (TA vs. TT).

**Figure 6:**
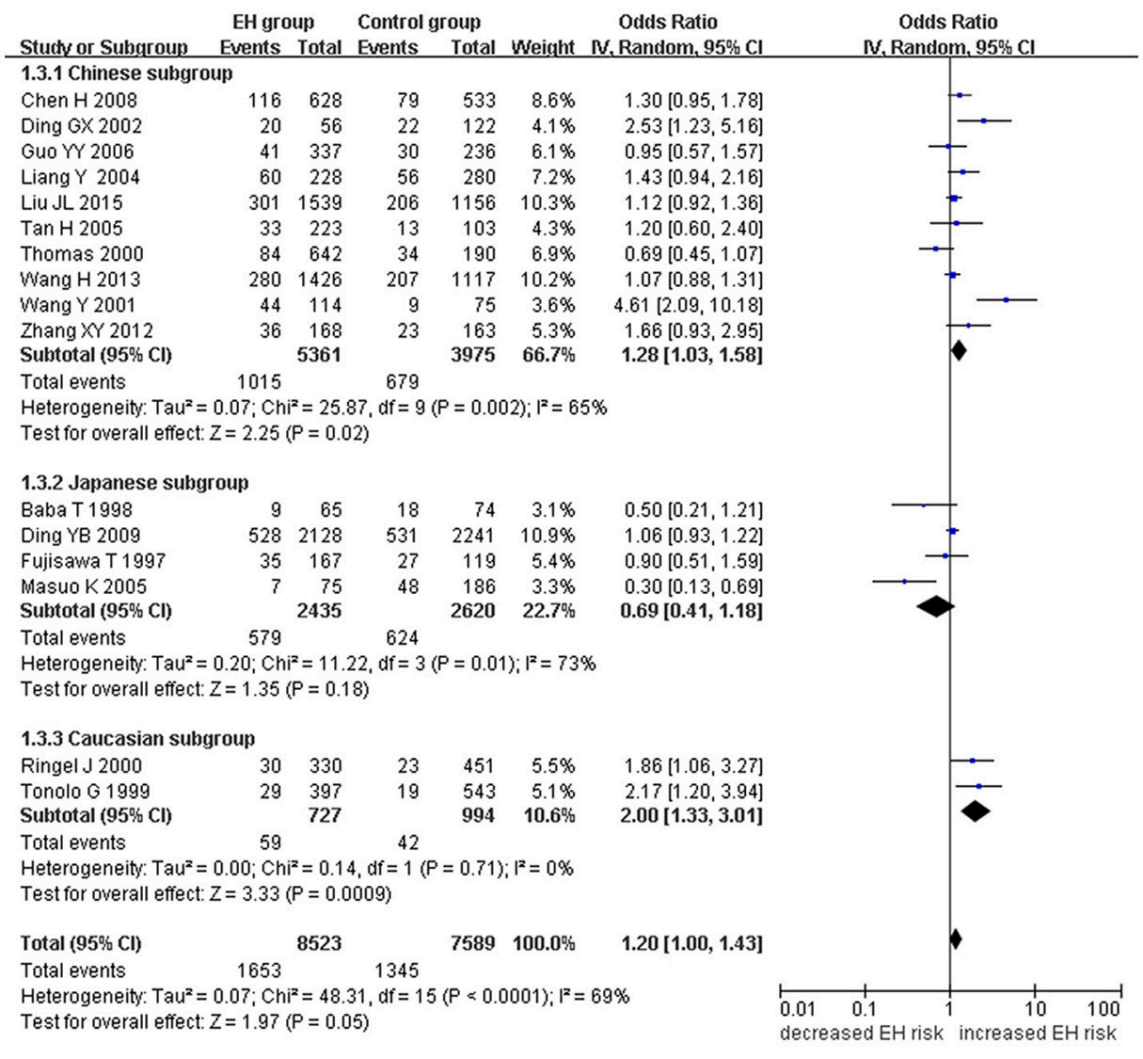
Forest plot of EH associated with *ADRB3* gene Trp64Arg polymorphism under an additive genetic model (A vs. T).

A marginally significant association between *ADRB3* gene Trp64Arg polymorphism and EH was found in the whole population under the additive genetic model (OR: 1.200, 95% CI: 1.00–1.43, *P* = 0.049). The Chinese subgroup showed a significant association under allelic (OR: 1.150, 95% CI: 1.002–1.320, *P* = 0.046), dominant (OR: 1.213, 95% CI: 1.005–1.464, *P* = 0.044), heterozygous (OR: 1.430, 95% CI:1.040–1.970, *P* = 0.03), and additive genetic models (OR: 1.280, 95% CI: 1. 030–1.580, *P* = 0.02). A significant association was also found in Caucasian subgroup under allelic (OR: 1.850, 95% CI: 1. 260–2.720, *P* = 0.002), dominant (OR: 2.004, 95% CI: 1.316–3.052, *P* = 0.001), heterozygous (OR: 2.220, 95% CI:1.450–3.400, *P* = 0.0002), and additive genetic models (OR: 2.000, 95% CI: 1. 330–3.010, *P* = 0.0009). (Table 2, Figures 1–6).

**Table 2:** Summary of meta-analysis of association between β*3-Adrenergic receptor (ADRB3)* gene Trp64Arg polymorphism and essential hypertension (EH).

**Genetic model**	**Pooled OR (95% CI)**	***Z*-value**	***P*-value**	**Study number**	**EH size**	**Control size**	**$P_{\text{HTG}\left(I_{\%}^{2}\right)}$**
Allelic genetic model	1.130 (0.990–1.280)	1.86	0.063	16	5,088	4,467	0.005^a^ (55.0%)
Chinese subgroup	1.150 (1.002–1.320)	1.99	0.046^a^	10	3,188	2,327	0.07 (43.0%)
Japanese subgroup	0.820 (0.570–1.160)	1.13	0.26	4	1,507	1,622	0.06 (59.0%)
Caucasian subgroup	1.850 (1.260–2.720)	3.13	0.002^a^	2	393	518	0.69 (0%)
Recessive genetic model	1.150 (0.890–1.480)	1.06	0.29	16	5,088	4,467	0.47 (0%)
Chinese subgroup	1.040 (0.730–1.470)	0.20	0.84	10	3,188	2,327	0.33 (13.0%)
Japanese subgroup	1.280 (0.890–1.860)	1.33	0.18	4	1,507	1,622	0.66 (0%)
Caucasian subgroup	NE			2	393	518	
Dominant genetic model	1.174 (0.985–1.400)	1.79	0.073	16	5,088	4,467	0.001^a^ (59.0%)
Chinese subgroup	1.213 (1.005–1.464)	2.02	0.044^a^	10	3,188	2,327	0.062 (44.6%)
Japanese subgroup	0.719 (0.427–1.210)	1.24	0.214	4	1,507	1,622	0.034^a^ (65.3%)
Caucasian subgroup	2.004 (1.316–3.052)	3.24	0.001^a^	2	393	518	0.718 (0%)
Homozygous genetic model	1.160 (0.900–1.500)	1.13	0.26	16	5,088	4,467	0.39 (6%)
Chinese subgroup	1.110 (0.780–1.580)	0.56	0.58	10	3,188	2,327	0.22 (24.0%)
Japanese subgroup	1.220 (0.840–1.770)	1.05	0.29	4	1,507	1,622	0.62 (0%)
Caucasian subgroup	NE			2	393	518	
Heterozygous genetic model	1.250 (0.950–1.650)	1.62	0.11	16	5,088	4,467	<0.00001^a^ (80.0%)
Chinese subgroup	1.430 (1.040–1.970)	2.21	0.03^a^	10	3,188	2,327	<0.0001^a^ (76.0%)
Japanese subgroup	0.520 (0.220–1.220)	1.50	0.13	4	1,507	1,622	0.0005^a^ (83.0%)
Caucasian subgroup	2.220 (1.450–3.400)	3.69	0.0002^a^	2	393	518	0.74 (0%)
Additive genetic model	1.200 (1.001–1.430)	1.97	0.049^a^	16	5,088	4,467	<0.0001^a^ (69.0%)
Chinese subgroup	1.280 (1.030–1.580)	2.25	0.02^a^	10	3,188	2,327	0.002^a^ (65.0%)
Japanese subgroup	0.690 (0.410–1.180)	1.35	0.18	4	1,507	1,622	0.01^a^ (73.0%)
Caucasian subgroup	2.000 (1.330–3.010)	3.33	0.0009^a^	2	393	518	0.71 (0%)

a *P < 0.05. CI, confidence interval; OR, odds ratio; EH size, the total number of EH cases; control size, the total number of control group; Allelic genetic model, A allele distribution frequency; Dominant genetic model, AA+TA vs. TT; Recessive genetic model, AA vs. TA+TT; Heterozygous genetic model, TA vs. TT; Homozygous genetic model, AA vs. TT; Additive genetic model, T vs. A. NE, not estimable*.

Significant HTG was detected under allelic, dominant, heterozygous, and additive genetic models (allelic: P_HTG_ = 0.005, *I*^2^ = 55.0%; dominant: P_HTG_ = 0.001, *I*^2^ = 59.0%; heterozygous: P_HTG_ < 0.00001, *I*^2^ = 80.0%; additive: P_HTG_ < 0.0001, *I*^2^ = 69.0%). Stratification by ethnicity, however, resulted in no significant HTG under the allelic genetic model in the Chinese (P_HTG_ = 0.07, *I*^2^ = 43.0%), Japanese (P_HTG_ = 0.06, *I*^2^ = 59.0%), or Caucasian subgroups (P_HTG_ = 0.69, *I*^2^ = 0%). Stratification significantly decreased in all of the three subgroups under the dominant, (Chinese: P_HTG_ = 0.062, *I*^2^ = 44.6%; Japanese: P_HTG_ = 0.034, *I*^2^ = 65.3%; and Caucasian subgroup: P_HTG_ = 0.718, *I*^2^ = 0%), the heterozygous (Chinese: P_HTG_ < 0.0001, *I*^2^ = 76.0%; Japanese: P_HTG_ = 0.0005, *I*^2^ = 83.0%; Caucasian subgroup: P_HTG_ = 0.740, *I*^2^ = 0%), and the additive genetic model (Chinese: P_HTG_ = 0.002, *I*^2^ = 65.0%; Japanese: P_HTG_ = 0.01, *I*^2^ = 73.0%; Caucasian subgroup: P_HTG_ = 0.71, *I*^2^ = 0%).

### Bias diagnostics

**Figure 7:**
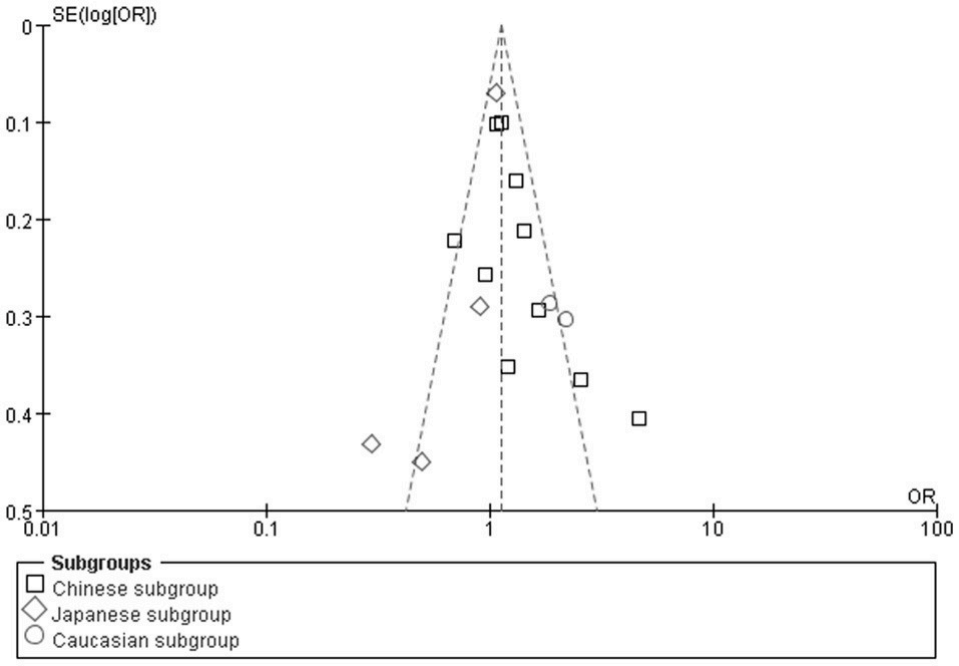
The funnel plot for the association of EH with *ADRB3* gene Trp64Arg polymorphism under an additive genetic model (A vs. T). The horizontal and vertical axis correspond to the OR and confidence limits. OR: odds ratio.

**Figure 8:**
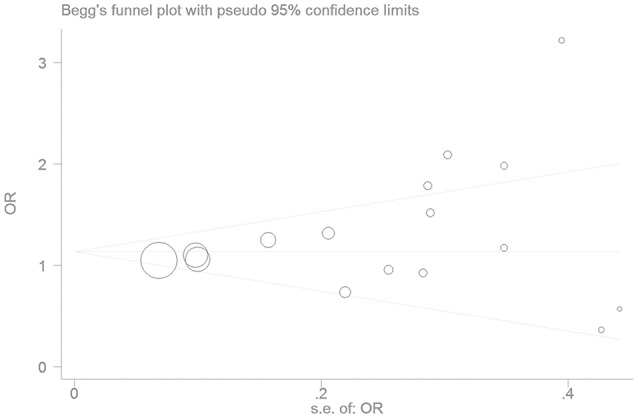
The Begg's funnel plot for the association of EH with *ADRB3* gene Trp64Arg polymorphism under an additive genetic model (A vs. T). The horizontal and vertical axis correspond to the OR and confidence limits. OR, odds ratio.

We found no publication bias in the funnel plot under the additive genetic model (Figure 7). In addition, there was no significant difference in the Egger's test yet, which implied that no publication bias existed in the present meta-analysis under the dominant genetic model (*T* = −0.86, *P* = 0.975) (Figure 8).

### Sensitivity analysis

**Figure 9:**
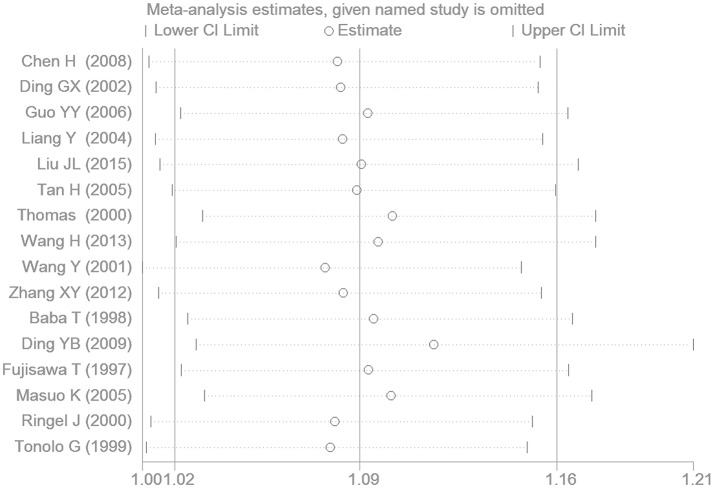
The sensitivity analysis plot for the association of EH with *ADRB3* gene Trp64Arg polymorphism under an additive genetic model (A vs. T).

The subsequent sensitivity analysis has been performed to observe whether the results are stable. After each study was excluded from the current meta-analysis, the significant was still significant under the additive genetic model (Figure 9).

## Discussion

A significant relationship was found in the present meta-analysis between *ADRB3* gene Trp64Arg polymorphism and EH under an additive genetic model (OR: 1.200, 95% CI: 1.00–1.43, *P* = 0.0049) in the whole population. In the Chinese subgroup, the association was more significant under allelic (OR: 1.150, 95% CI: 1.002–1.320, *P* = 0.046), dominant (OR: 1.213, 95% CI: 1.005–1.464, *P* = 0.044), heterozygous (OR: 1.430, 95% CI:1.040–1.970, *P* = 0.03), and additive genetic models (OR: 1.280, 95% CI: 1.030–1.580, *P* = 0.02). An increased association was also found in Caucasian subgroup under allelic (OR: 1.850, 95% CI: 1. 260–2.720, *P* = 0.002), dominant (OR: 2.004, 95% CI: 1.316–3.052, *P* = 0.001), heterozygous (OR: 2.220, 95% CI: 1.450–3.400, *P* = 0.0002), and additive genetic models (OR: 2.000, 95% CI: 1. 330–3.010, *P* = 0.0009).

The fixed model was used for the recessive or homozygous genetic models because no significant heterogeneity was detected (P_HTG_ > 0.05). Although there was significant HTG under the allelic, dominant, heterozygous, and additive genetic models (P_HTG_ < 0.05), it was reduced significantly when stratified by ethnicities, lending further strength to the positive association between the polymorphism and EH. In addition, the average 64Arg minor allele frequency is 0.16, 0.18, 0.06 in the Chinese, Japanese, and Caucasian populations. The minor allele frequencies of the polymorphism is different across different populations. The lower the minor allele frequency, the more significant the association of this mutation and EH. It might explain why the OR is diluted when assessing the whole sample as compared to only the Chinese or Caucasians. As six genetic models were used, only the analysis using additive genetic model was identified a prior and primary analysis. Other analyses under the allele, recessive, dominant, heterozygous, and homozygous genetic models were listed as secondary analyses.

EH is one of the most common cardiovascular conditions and its morbidity has increased annually in China recently. EH increases a patient's risk of coronary artery disease (CAD), stroke, cardiovascular disease, and renal injury. Among the many risk factors involved in the pathogenesis of EH, IR, and dyslipidemia are likely the most important. EH is almost always accompanied by CAD, obesity, hyperlipemia, and/or DM. Reaven et al. call it X syndrome. The central links are IR and dyslipidemia which are considered to have the common hereditary basis (Reaven and Chen, 1988).

The ADRB3 receptor is widely distributed throughout human adipose tissue. Trp64Arg polymorphism is the only functional variant of ADRB3 protein. It has been found that this polymorphism has a significant influence on peak cAMP levels upon excitement with a selective β-receptor agonist, and receptor down-regulation induced by other agonists (Piétri-Rouxel et al., 1997). The *ADRB3* 64Arg carriers have the *ADRB3* gene expression defects and abnormal ADRB3 protein structure which lead to the decreased ADRB3 protein function. The impaired and inhibited cellular signal transduction caused the decreased visceral fat lipolysis and energy production which contributed to an increased body weight and a reduced basal metabolic rate (Guay et al., 2014; De Luis Román et al., 2017). The body mass was thus increased and the norepinephrine's sensitivity was improved, and the blood pressure was elevated (Baba et al., 1998).

In 2010, the meta-analysis by Kitsios et al. found a significant association for the Trp64Arg variant of the *ADRB3* gene and EH only under the dominant model (OR: 1.31, 95% CI: 1.07–1.60), not under the allele contrast model (OR: 1.00, 95% CI: 0.83–1.21) (Kitsios and Zintzaras, 2010). While the Kitsios analysis only included 6 studies or 13 studies in HWE under the allele or dominant respectively, the current meta-analysis included 16 studies in HWE under six genetic models and may therefore present a more objective perspective. In addition, stratification by ethnicity may make this analysis more comprehensive than that by Kitsios et al.

The PRISMA workflow was followed by the current meta-analysis (Page and Moher, 2017). Our analysis, however, does not substitute for a large-scale or prospective study on the relationship of *ADRB3* gene Trp64Arg polymorphism and EH. Serum ADRB3 levels can be influenced by a number of factors. such as obesity, DM, and hyperlipemia. The micro-effects of other genes combined may additionally influence an individual's susceptibility for EH. Polymorphisms in other genes, such as *angiotensin converting enzyme* gene insertion/deletion polymorphism, α-Adducin Gly460Trp gene mutation, bradykinin β2 receptor−58T/C gene polymorphism, TNF-α G308A gene polymorphism may additionally influence the EH susceptibility (Li, 2012a,b; Li et al., 2012; Li Y., 2012).

In brief, *ADRB3* gene Trp64Arg polymorphism was significantly correlated with increased EH risk, especially in the Chinese and Caucasian population. Carriers of the Arg allele of *ADRB3* gene Trp64Arg polymorphism may be at an increased risk of EH. This conclusion maybe helpful in the formulation of a novel personalized EH treatment approach. Considering the limitations mentioned above, more researches on the association of *ADRB3* gene Trp64Arg polymorphism and EH are needed to further confirm the conclusions.

## Author contributions

YL and XL: Conceived and designed the experiments; YL and HW: Performed the experiments; YL and YZ: Analyzed the data; YL: Contributed reagents, material, and analysis tools; YL: Wrote the manuscript; YL and XY: Reference collection and data management; YL, HG, HK, and GG: Statistical analyses and paper writing; YL: Study design.

### Conflict of interest statement

The authors declare that the research was conducted in the absence of any commercial or financial relationships that could be construed as a potential conflict of interest.

## Supplementary material

Supplementary Table 1 PRISMA Checklist.

Supplementary Table 2 PRISMA Flow Diagram.
