# Supplementary material for: ADRB3 Gene Trp64Arg Polymorphism and Essential Hypertension: A Meta-Analysis Including 9,555 Subjects
Source: Front Genet. 2018 Apr 4;9:106. doi: 10.3389/fgene.2018.00106 (PMC5893850; doi:10.3389/fgene.2018.00106)
Supplement: Supplementary Table 2 — PRISMA Flow Diagram. [file Table2.DOCX]

**PRISMA 2009 Flow Diagram**


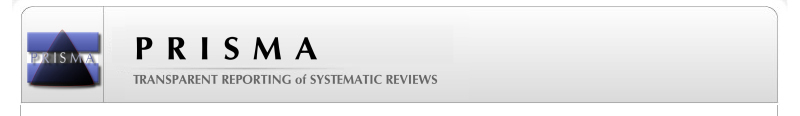


Records excluded for repeated publication
(n = 0 )

Records excluded for no association with *ADRB3* gene Trp64Arg polymorphism or EH

(n =2)

Full-text articles excluded for deviation from HWE (n =4)

Records excluded for review characteristic
(n =3 )

Studies included in qualitative synthesis
(n =16)

Full-text articles assessed for eligibility
(n =18)

Records screened
(n =22 )

Records after duplicates removed
(n =25)

Additional records identified through other sources
(n =0 )

## Identification

## Eligibility

## Included

## Screening

Records identified through database searching
(n =25 )
